# The complete chloroplast genome of *Quercus fenchengensis* and the phylogenetic implication

**DOI:** 10.1080/23802359.2019.1666040

**Published:** 2019-09-20

**Authors:** Hua-Lei Hu, Lian-Zhen Wang, Jian Yang, Ru-Song Zhang, Qun Li, Yan-Qun Liu, Li Qin

**Affiliations:** a Department of Sericulture, College of Bioscience and Biotechnology, Shenyang Agricultural University, Shenyang, Liaoning, China;; b Research Group of Oaks, Sericultural Institute of Liaoning Province, Fengcheng, Liaoning, China

**Keywords:** *Quercus fenchengensis*, chloroplast genome, phylogenetic relationship

## Abstract

*Quercus fenchengsis* is a rare Chinese oak species sporadically recorded in Fengcheng, Liaoning, Yuntai Mountain, Henan, and Qinling Mountains, Shaanxi. Here, the complete chloroplast (cp) genome of *Q. fenchengensis* was first reported. The cp genome is 161,296 bp in length with a GC content of 36.81%, and encodes 134 genes (86 protein-coding genes, 40 tRNA genes and eight rRNA genes). The phylogenetic tree based on the cp genome confirmed that *Q. fenchengensis* has a close relationship with *Quercus aliena acutiserrata* and *Quercus dentata*, consistent with the previous morphology-based suggestion that it would be a hybrid of *Q. aliena acutiserrata* and *Q. dentata*.

*Quercus fenchengensis* is a Chinese oak first recorded in Fengcheng, Liaoning (Jen et al. 1984), and then found in Yuntai Mountain, Henan and Qinling Mountains, Shaanxi. Morphologically, the nut and shell look like the *Quercus dentata*, but with short bracteoles; the branches and leaves resemble the *Quercus aliena acutiserrata*, but with sparse stelatehairs on the underside of the leaf (Jen et al. 1984). It has been suggested that it may be the hybrid of *Q. aliena acutiserrata* and *Q. dentata* (Xu and Ren 1998; Huang et al. 1999). So far, the molecular information regarding this rare oak species is severely limited. In this study, we report the complete chloroplast (cp) genome of the species to provide useful genetic information for this species.

The leaves of *Q. fenchengensis* was collected from a single individual tree at the oak field (N40°28′13.69″; E123°59′17.07″) of Sericultural Institute of Liaoning Province, Fengcheng, China. The specimen was stored with the collection number of OAK_FEN_01 at Department of Sericulture, Shenyang Agricultural University, China. Chloroplast DNA extraction and next-generation sequencing were conducted with an Illumina Hiseq 2500 platform by Personal Biotechnology (Shanghai, China). A reference-guided genome assembly was performed with the cp genome of *Q. aliena* (KU240007; Yang et al. 2016) as the reference. We used the program DOGMA to annotate the cp genome (Wyman et al. 2004) and compared them with the published cp genomes of *Quercus* species for correction manually. The cp genome sequence has been deposited at Genbank with accession number MN095295.

The cp genome of *Q. fenchengensis* is 161,296 in size, forming a typical quadripartite structure as known oaks. The circular cp genome is composed of two single-copy regions separated by a pair of inverted repeats [large single-copy region (LSC): 90,584 bp; small single-copy region (SSC): 19,058 bp; inverted repeats (IRs): 25,827 bp]. The cp genome encodes 134 genes, including 86 protein-coding genes, 40 tRNA genes, and 8 rRNA genes. There are 22 intron-containing genes in this cp genome, including 14 protein-coding genes and 8 tRNA genes. The two *ycf1* genes stretch across the IR/SSC borders.

The cp genome sequence of *Q. fenchengensis* together with other 14 *Quercus* species was used to reconstruct the phylogenetic relationships of oaks with Bayesian inference (BI) method under the GTR + G + I model by MrBayes (Huelsenbeck and Ronquist 2001). In the phylogenetic tree (Figure 1), the majority of phylogenetic branches are supported by the high posterior probabilities. The phylogenetic analysis based on the cp genome confirmed that *Q. fenchengensis* has a close relationship with *Q. aliena acutiserrata* followed by *Q.dentata*. This result is in line with the previous morphological characters-based suggestion that *Q. fenchengensis* would be a hybrid of *Q. aliena acutiserrata* and *Q. dentata* (Xu and Ren 1998; Huang et al. 1999). However, comparison of the cp genome sequence between *Q. fenchengensis* and *Q. aliena acutiserrata* revealed 290 single nucleotide variants (SNVs). Thus, to address the evolutionary origin of *Q. fenchengensis*, more samplings of oak species and nuclear gene markers need to be covered.

**Figure 1.**
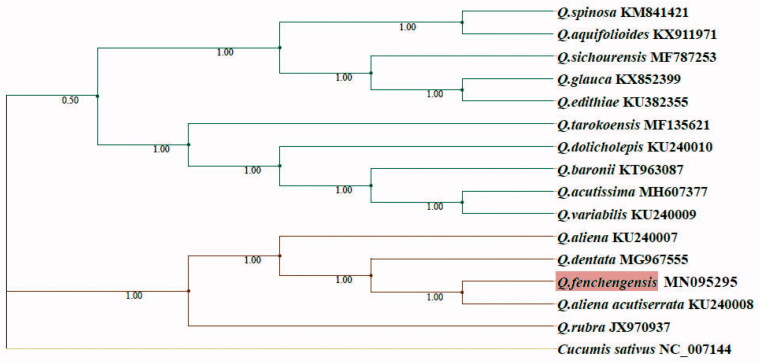
Phylogeny of 15 *Quercus* species based on whole chloroplast genomes. BI tree was built with Bayesian inference method under the model of GTR + G + I. The accession numbers are listed following species name. Bayesian posterior probability is given at each node.
